# Ear Acupressure for Allergic Rhinitis: A Systematic Review and Meta-Analysis of Randomized Controlled Trials

**DOI:** 10.1155/2021/6699749

**Published:** 2021-05-03

**Authors:** Juan Zhong, Shuqin Liu, Dan Lai, Tao Lu, Yifeng Shen, Qisheng Gong, Peijia Li, Qinxiu Zhang

**Affiliations:** ^1^Hospital of Chengdu University of Traditional Chinese Medicine, No. 39 Shi-er-qiao Road, Chengdu 610072, Sichuan Province, China; ^2^Department of Otolaryngology Head and Neck Surgery, The Affiliated Hospital of Southwest Medical University, No. 25 TaiPing Street, Luzhouv 646000, Sichuan, China; ^3^Otolaryngology and Head & Neck Surgery Department One, First Affiliated Hospital of Kunming Medical University, Kunming 650032, Yunnan, China; ^4^School of Medical and Life Sciences/Reproductive & Women-Children Hospital, Chengdu University of Traditional Chinese Medicine, Chengdu 610072, Sichuan Province, China

## Abstract

**Background:**

The treatment effects and safety of ear acupressure (EAP) for patients with allergic rhinitis (AR) have yet to be clarified.

**Objective:**

To evaluate the effects and safety of EAP in AR patients.

**Design:**

Systematic review of published studies.

**Methods:**

A total of 24 English and Chinese databases (PubMed, EMBASE (Excerpta Medical Database), Cochrane Central Register of Controlled Trials, CINAHL, Informit, ScienceDirect, LILACS (Latin American and Caribbean Health Sciences), ProQuest, AMED, Blackwell Synergy, PsycINFO, Panteleimon, AcuBriefs, KoreaMed, IndMed, Ingenta, mRCT, ISI Web of Knowledge, ERIC, VIP Information (http://www.cqvip.com), China National Knowledge Infrastructure (http://www.cnki.net), Cochrane Library, Chinese Cochrane Centre Controlled Trials Register Platform, and Wanfang Chinese Digital Periodical and Conference Database) were searched from their respective inceptions to August 2020 to collect randomized controlled trials of ear acupressure for allergic rhinitis. We performed literature inclusion, data extraction, and trial quality evaluations. Methodological quality was assessed according to the Cochrane Handbook. Revman5.3 was used for all analyses.

**Results:**

A total of 203 trials were identified and eleven studies involved 1094 participants aged 3–70 years. EAP was better than control group interventions in terms of effectiveness (risk ratio (RR): 0.51; 95% confidence interval (CI): 0.36–0.70; *P* < 0.0001). EAP was superior to sham EAP in terms of improvement of the total nasal symptom score (RR: −0.50; 95% CI: −0.96–0.05; *P* = 0.03), sneezing score (RR: −0.36; 95% CI: −0.59–0.12; *P* = 0.003), global QoL score (RR: 0.42; 95% CI: 0.04–0.08; *P* = 0.03), and eye symptom score (RR: −0.36; 95% CI: −0.67–0.05; *P* = 0.02).

**Conclusions:**

Despite the positive results, it is premature to confirm the efficacy of EAP for treating AR. More high-quality studies are needed to confirm safety and efficacy.

## 1. Introduction

Allergic rhinitis (AR) is a global health problem. It is a common symptomatic, inflammatory, and immunological disorder of the nasal mucosa, characterised by four classic symptoms: sneezing, nasal itching, airflow obstruction, and clear nasal discharge caused by IgE-mediated reactions [1]. AR can be subdivided into intermittent (four symptoms for <4 days per week or for <4 weeks) and persistent disease (four symptoms for >4 days per week or for >4 weeks) [2].

A self-reported epidemiologic study suggested that 10–30% of adults have AR and no fewer than 40% of children have AR [3]. In other words, more than 60 million people suffer from AR in the United States annually. A European epidemiologic study reported a prevalence rate of 25% [4].

Due to the annoying symptoms, AR has significant adverse effects according to several trials; these include disturbing symptoms [5–9], alterations in quality of life (QOL) [5, 8, 10–15], hindrance of daily activities [16–20], emotional disturbances [9, 21], sleep [10, 22–26], and education disturbances [9, 16, 27]. These data suggest that the effect on adolescent life is negative and far reaching. AR has been shown to be associated with obstructive sleep apnoea [27].

Avoidance of exposure to specific allergens, patient education, pharmacological treatment, and immunotherapy form the current management approaches. Among these methods, medications are the most often selected strategy. A stepwise medical treatment protocol was proposed at the ARIA workshop report [28]. However, treatment based on guidelines is not effective in all patients [29]. Hence, many allergic rhinitis sufferers seek complementary and alternative medicine (CAM).

EAP is an alternative therapy in which magnetic bead or the seed of cowherb is attached to specifically stimulate points on the pinna. From the point of view of traditional Chinese medicine (TCM), all the major energy lines (meridians where acupuncture points are situated) are directly or indirectly connected to the ear. EAP was a shown to be effective for relief AR symptoms [30, 31]. However, a previous meta-analysis suggested that the benefit of ear acupressure for symptomatic relief of allergic rhinitis is unknown [32].

In this previously published meta-analysis, the authors summarized the evidence of EAP on AR. However, this study made a mistake in literature, including a duplicate publication of Rao et al. (2005) and Rao and Han et al. (2006). In their results section, the authors stated that “ear acupressure was more effective than herbal medicine, as effective as body acupuncture or antihistamine for the short-term effect, but it was more effective than antihistamine for the long-term effect.” This statement may have been exaggerated, and the quality of evidence was low. In the present systematic review and meta-analysis, we provide an updated summary of evidence to evaluate the safety and efficacy of EAP for patients with AR.

## 2. Methods

Methodologic issues were resolved with guidance from the Cochrane Handbook for Systematic Reviews of Interventions [33].

### 2.1. Search Strategy

A total of 24 English and Chinese databases were searched from their inceptions to August 18, 2020. These were PubMed, EMBASE (Excerpta Medical Database), Cochrane Central Register of Controlled Trials, CINAHL, Informit, Science Direct, LILACS (Latin American and Caribbean Health Sciences), ProQuest, AMED, Blackwell Synergy, PsycINFO, Panteleimon, AcuBriefs, KoreaMed, IndMed, Ingenta, mRCT, ISI Web of Knowledge, ERIC, VIP Information (http://www.cqvip.com), China National Knowledge Infrastructure (http://www.cnki.net), Cochrane Library, Chinese Cochrane Centre Controlled Trials Register Platform, and Wanfang Chinese Digital Periodical and Conference Database. The Chinese Clinical Trial Registry Centre was also retrieved for ongoing trials. References of related identified publications were checked for additional trials, and we contacted authors by e-mail or telephone for additional data where necessary.

Throughout the search process, the following key words were used: the combination of allergic, rhinitis, rhinallergosis, AR, allergy, rhinitis, hay fever, ear, acupressure, acupuncture, auricular, acupoint, sticking, randomized controlled trial, RCT, and their synonyms. Two authors (SQL and QXZ) screened all citations independently.  Table 1 displays the search strategy of the Cochrane Library.

### 2.2. Study Selection

#### 2.2.1. Eligible Criteria

Randomised controlled trials of EAP for AR were taken into account regardless of language or publication year. Patients presenting with seasonal AR or perennial AR of any age or gender were all included. We compared EAP with conventional therapy or Chinese herbal medicine formula or acupuncture or electroacupuncture or surgical therapy or placebo regimens studies.

#### 2.2.2. Ineligible Criteria

Observational studies, case reports, case series, letter, qualitative studies, and uncontrolled studies were excluded. Quasi-RCTs are not truly RCT. Including quasi-RCTs in the review may be detrimental to the power of conclusion. Hence, quasi-RCTs were also excluded. Allergic rhinitis merged with allergic asthma or allergic conjunctivitis and other allergic diseases were excluded. This was performed because targeted drug combination methods in these studies could not be used to compare effects.

EAP as intervention in the control group was excluded. When EAP was compared with other types of CHMFs or some other alternative therapy such as moxibustion therapy, nose massage, plaster therapy, or acupoint injection as the intervention treatment group, these were excluded due to the idea that combination therapy would disturb the efficacy summary. EAP as intervention in two groups using different auricular points was also excluded because these studies can be identified as explorations for the stimulation effect of various auricular ear points. Diagnostic criteria were required because accurate diagnosis is a prerequisite for targeted treatment.

All titles and abstracts of identified studies were initially scanned independently by our two authors (JZ and SQL). The full-text articles were obtained for further screening for inclusion in this review by these two authors when needed. A determination was then made as to whether the studies met our inclusion criteria. Any conflicts or disagreement were resolved by discussion.

### 2.3. Outcome Measures

Trials were required to include as outcome measures either relief of symptoms of AR or evaluation of the efficacy of EAP in AR. The efficacy of EAP for AR was set as primary outcomes. Improvement in quality of life, improvement of symptom scoring and other scale, and adverse events were set as secondary outcomes.

### 2.4. Methodological Quality Assessment, Data Extraction, and Data Analysis

The risk of bias was assessed according to the Cochrane Handbook for Systematic Reviews of Interventions. The latest version of this tool was updated in March 2011, version 5.1.0 (http://www.handbook.cochrane.org/). Risk of bias items included the following: randomization sequence generation, allocation concealment, blinding of participants or healthcare providers, detection bias, incompleteness bias, reporting bias, and other biases. Raw data of all included studies containing the details of authors, the publication information, and design information of the original study were separately extracted by three authors (DL, TL, and QG).

### 2.5. Data Analysis

Review Manager software version 5.3 was used to pool our data to perform the meta-analysis. Risk ratio (RR) was chosen for dichotomous data (efficacy, recurrence rate, and adverse events). Confidence interval (CI) was set at 95%, and *P* < 0.05 was defined as statistically significant. Cochrane *X*^2^ and *I*^2^ tests were used to investigate the heterogeneity of data. The statistical heterogeneity was considered substantial when *P* < 0.05 and *I*^2^ > 50%, and then, a random effect model was used to calculate the effect size. If *P* > 0.05 and *I*^2^ < 50%, then the studies included were homogeneous, and a fixed effect model was applied. Sensitivity analysis was conducted to test whether the resulting war was robust by excluding the study one by one and comparing the rest of the studies' effects with all the studies' total effects. We pooled trials when the intervention forms of those studies were adequately similar. Specific subgroups were analysed according to similar intervention forms or similar design. Funnel plots were generated for more than ten studies.

## 3. Results

**Figure 1:**
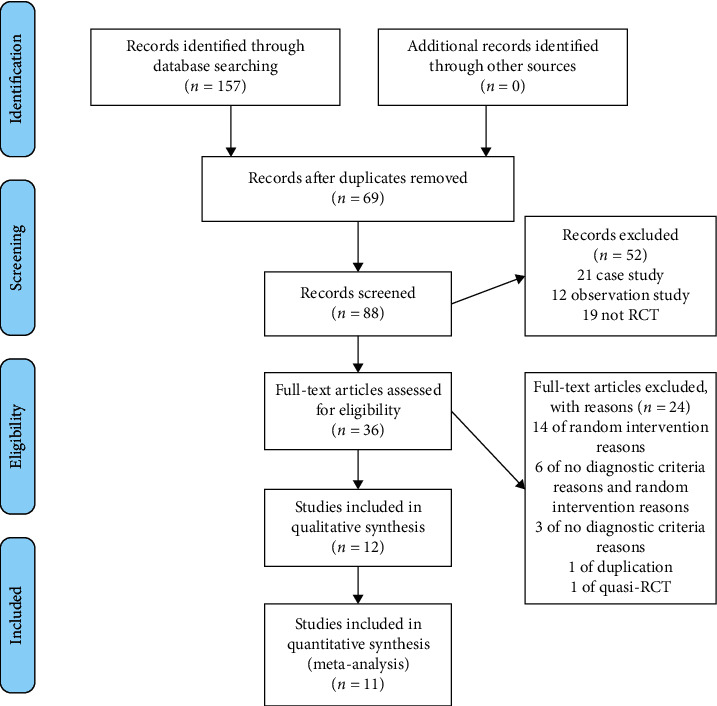
Flowchart of database searching and study identification.

We initially identified a total of 157 trials using the specific search strategy. No unpublished or ongoing studies were found. Sixty-nine duplicated texts were excluded. After reviewing titles, abstracts, and keywords, 52 studies were excluded for failure to conform to inclusion criteria. Thirty-six studies had initially appeared to meet our inclusion criteria. After the full texts were read, 25 were excluded, and *t* studies finally met our inclusion criteria. The study selection process is outlined in Figure 1.

### 3.1. Characteristics of Included Studies

There were eleven studies total. These included 10 Chinese language studies and one English language study [32], comprising 1094 participants aged 3–70 years [32, 34–43], published between 2005 and 2019. All Chinese studies come from the Chinese mainland.

Interventions in five Chinese studies [34, 35, 37, 38, 43] were EAP versus CT. Four studies [34, 35, 38, 43] compared EAP to CT, including levocabastine, beclomethasone propionate nasal spray, astemizole, and cetirizine [37]. One study compared EAP combined with cetirizine to cetirizine alone [37]. Among these studies, Rao et al. included three comparisons, that is, the experiment group (ear-acupressure) and two control groups (acupuncture and cetirizine). In order for the reader to accurately identify intervention data, we extracted cetirizine control group data from Rao et al. (2005a). There are 245 participants in Claire et al. (2014). This was an international, multicentre, randomized, single-blind, sham-controlled trial. The intervention was EAP vs. sham EAP. Acupuncture therapy was the intervention of the control group in three studies [34, 36, 40], and electroacupuncture was the intervention of the control group in one study [39]. In the remaining two studies, CHMF was the intervention in the control group; these were Xinqin granules [41] and Shetizhiqiu decoction [43].

In these studies, Semen vaccariae (cow soapwort seed or Wang Bu Liu Xing) were used in eleven studies [34, 38–43] to press the ear points, while stainless steel pellets were used by Claire et al [38].

**Figure 2:**
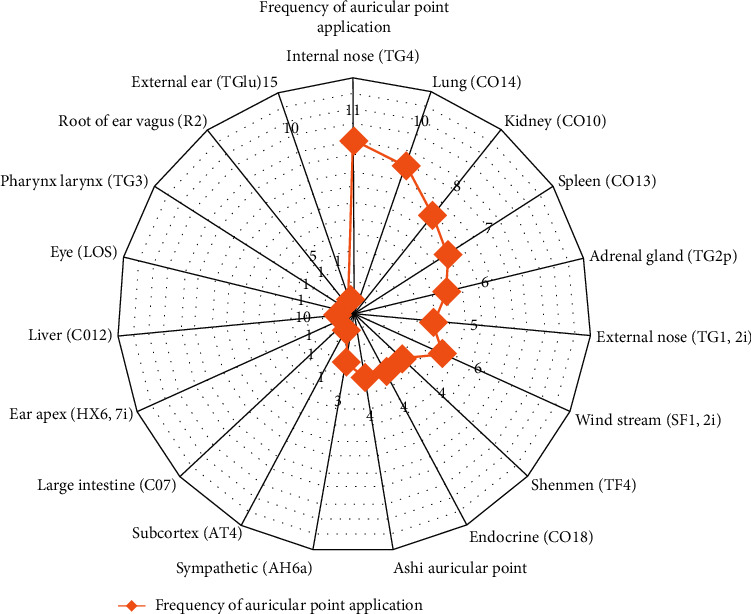
Frequency of auricular point application.

All studies provided the details of points used for ear acupressure. Among the total 19 ear points used in these 11 studies, Nei Bi (TG4) was used in all the studies. The lung (CO14), kidney (CO10), spleen (CO13), and adrenal gland (TG2p) were applied 10, 8, 7, and 6 times, respectively. We presented the frequency of ear point use in a radar plot (Figure 2).

Referring to outcome measures, the effective rate indicator was regarded as the most important outcome measure in 10 trials [34–42]. Total nasal symptom score was calculated in two trials [37, 44]. The visual analogue scale was used in one trial [37]. The Quality Of Life Questionnaire is used only one study [39]. Of these 12 studies, five did not report details of adverse events [35, 36, 38–40], and six trials [32, 34, 37, 41, 42] recorded adverse events. In Claire et al., adverse events (AEs) were a secondary outcome. Characteristics of included studies are displayed in Table 2.

### 3.2. Reasons for Study Exclusion

After our two authors read full texts of all included studies, we excluded 25 trials. The reasons for exclusion of those studies were as follows:

1. If a trial did not mention “diagnostic criteria,” we excluded it on the basis of having “diagnostic criteria reason”
2. If a trial did not meet inclusion criteria for interventions, we excluded it on the basis of “intervention reason”
3. If a study was a repeated publication, we excluded it on the basis of being “duplication”

Overall, there were ten “diagnostic criteria reason” studies, 14 “intervention reason” trials, and six that had both. Those were excluded for incomparability of the interventions. One “duplication” was identified. Characteristics of the 25 excluded studies are displayed in Table 3.

A list of 25 excluded studies by reading full text is displayed in Table 4.

### 3.3. Methodological Quality of Included Studies

#### 3.3.1. Allocation (Selection Bias)

In 11 included studies, there were all designed as randomized controlled trials [32, 34–43].

In six RCTs [35–38, 40, 42], the method of randomization and allocation was not mentioned. Because of the lack of allocation concealment and the details of the randomization procedure, there might be a high risk of selection bias in these trials.

In the study by Lu et al. (2015) [39], randomization and the random allocation sequence concealment were reported. Hence, a low risk of bias could be defined in these trials.

Three studies conducted by Li et al. (2018) [42], Rao et al. (2005) [34], and Liao et al. (2016) [41] mentioned that a random number table tool was used for the allocation of participants. However, in the original texts, the authors failed to clearly describe the details regarding how the participants were allocated and concealed. Therefore, there was a high risk of selection bias in these studies.

#### 3.3.2. Blinding (Performance and Detection Biases)

Of the 11 studies, only Claire et al. (2014) [32] and Lu et al. (2015) [39] presented blinding information. In Claire et al. (2014), randomization numbers were reported as generated by an independent statistician using a computer system and sealed in individual opaque envelopes. In the study Lu et al. (2015) [39], to blind the outcome assessor, researchers who knew the whole course of treatment were not involved. In this way, the risk of performance and detection bias were be classified as low.

The remaining 9 trials failed to mention their blinding [34–38, 40–42]. Perhaps blinding was difficult because the materials and manipulations used in the treatment were totally different in the test and control groups. However, at least the outcome assessors should have been blinded. Because there was no blinding in these studies, there might be high risk of performance and detection biases.

#### 3.3.3. Incomplete Outcome Data (Attrition Bias)

In the study by Claire et al. (2014) [32], the sample size was determined based on previous reported results. Intention-to-treat analysis and dropout information were offered in this study also. Therefore, there might be a low risk of attrition bias.

Information regarding patient withdrawal was provided in Rao et al. (2005) [34], Liao et al. (2016) [41], and Zhao et al. (2019) [43]. Zhao et al. [43] reported one participant dropout into both groups. Liao et al. [41] reported two dropouts in the control group; however, the reason why those two patients were dropped out was not noted. Rao et al. [34] reported that three in the acupuncture therapy group (fear of pain), one in the ear-acupressure group (refused to continue treatment because of pain in the pinna), and four in the control group (severe headache, drowsiness and other adverse reactions occurred during taking the medicine could not continue to receive treatment) were dropped out. Therefore, those three studies were at a low risk for incomplete outcome reporting bias. However, a crucial limitation was that intention-to-treat and per-protocol analyses were not conducted in these three trials.

There were some limitations in seven studies (Ouyang et al. (2012), Yuan et al. (2013), Li et al. (2018), Ye et al. (2008), Yuan et al. (2016), Han et al. (2006), and Lu et al. (2015)) [35–40, 42] in terms of incompleteness bias because there were no sample size calculations, and no cases were reported to have been lost to follow-up or withdrawn from the trials. The incompleteness bias might be unclear in these trials due to failure to report dropouts.

#### 3.3.4. Selective Reporting (Reporting Bias)

To reduce reporting bias, all analyses with and without statistically significant differences should be reported. One of the ways to assess reporting bias is to compare the results in the final reports with those in the protocol. However, 10 in 11 studies protocol could not be found in these studies, and only Claire et al. (2014) [32] declared a clinical trial registration number (ACTRN12608000149369). It was difficult to determine whether the other 10 outcomes were included in the published reports. Hence, the risk of reporting bias in these 10 studies was classified as “unclear,” while the risk of reporting bias in Claire et al. (2014) [32] was low.

#### 3.3.5. Other Potential Sources of Bias

**Figure 3:**
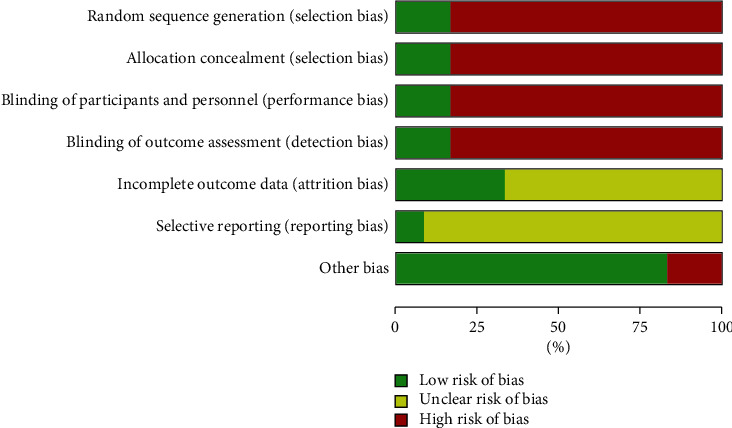
The risk of bias graph of authors' judgements concerning included studies.

**Figure 4:**
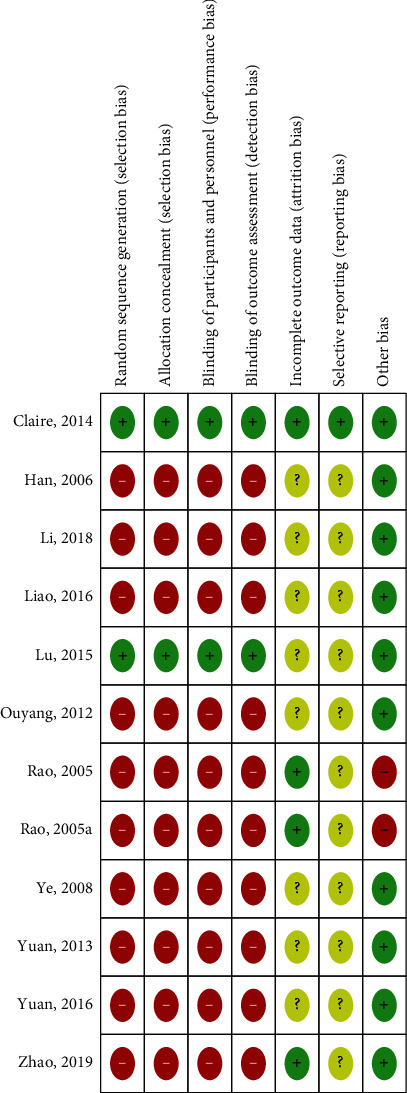
The risk of bias summary of authors' judgements concerning included studies.

We set “support from pharmaceutical manufacturers” as other potential source of bias. Support in the form of free medical supplies, research funding support, and medical supplies marketing cooperation coming from pharmaceutical manufacturers could bias results. Nevertheless, no other potential sources of bias were found in these studies. There was publication bias by Rao et al. (2005). The risk of bias graph and summary of authors' judgements concerning included studies are shown in Figures 3 and 4, respectively.

## 4. Outcomes

### 4.1. Clinical Effectiveness

According to raw data extracted from 11 original texts, primary outcomes including effective rate, total nasal symptom score, runny nasal score, sneezing score, global QoL score, and eye symptoms were presented. Adverse events were presented as secondary outcomes.

### 4.2. Primary Outcomes

#### 4.2.1. Total Effective Rate of EAP for AR

The effective rate is a composite endpoint composed of improvement of clinical symptoms. The results can be divided into three categories: significantly effective, effective, and ineffective. Four versions of the Chinese AR Clinical Handbook Indicator recommend AR therapies [46–49]. They were slightly different. In Haikou, 1997 criteria [46], “according to the sum of symptoms and signs scores before and after treatment, the efficacy of perennial allergic rhinitis was evaluated by the following formula: ≥51% was considered effective, 50%–21% was considered effective, and ≤20% was considered ineffective.” In other reports, “according to the symptoms and signs score, the efficacy was evaluated by the following scoring methods: ≥66% was marked as effective, 65%–26% as effective, and ≤25% as ineffective.”

In our review, there are 10 trials reporting effectiveness rates [34–43]. Han et al. 2006 and Rao et al. 2005 applied 1997 criteria, different from the others. We divided these studies into six subgroups according to intervention comparison forms into two groups: (1) four studies (Han et al., 2006; Li et al., 2008; Rao et al., 2005a; and Yuan et al., 2013) were EAP vs. CMT (conventional medicine therapy) comparisons; (2) Rao et al. (2005) used EAP vs. acupuncture; (3) two trials (Ye et al., 2008 and Yuan et al., 2016) used EAP plus acupuncture vs. acupuncture alone; (4) Lu et al. studied EPA plus electroacupuncture vs. electroacupuncture alone; (5) EPA plus Chinese medicine formula (CMF) vs. Chinese medicine formula alone was used in two articles (Liao et al. 2016; Zhao et al. 2019); and (6) one trial (Ouyang et al. 2012) studied EAP plus conventional therapy (CT) vs. CT alone.

**Figure 5:**
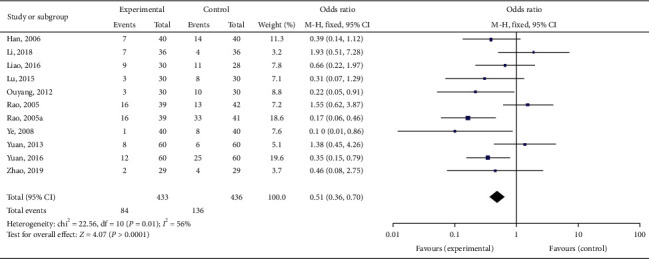
Forest plot for total effective rate.

**Figure 6:**
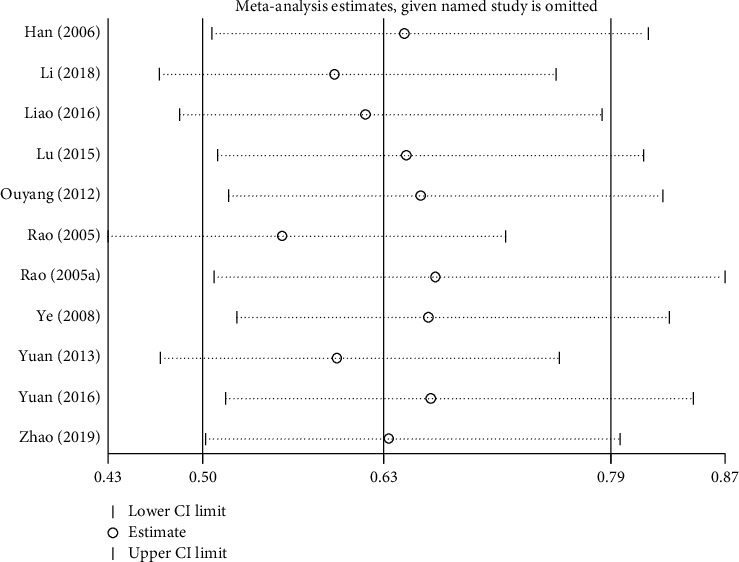
Sensitivity analysis for total effective rate.

We pooled these trials using RevMan 5.3. A total of 869 participants were pooled, 433 in the treatment group and 436 in the control group. We used a random-effects model because of the significant heterogeneity (*I*^2^ = 56%, *P* = 0.01). Meta-analysis revealed that the total effective rate in the treatment group was greater than that in the control group (pooled risk ratio = 0.51, 95% CI (0.36–0.70), *P* < 0.0001; Figure 5). Sensitivity analysis indicated that their result was robust (Figure 6).

**Figure 7:**
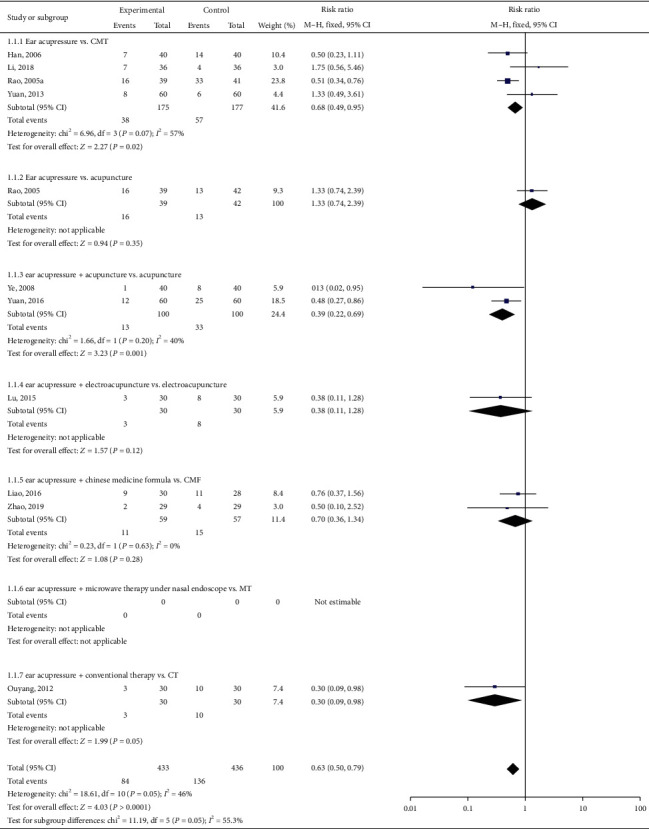
Forest plot for subgroup of total effective rate.

Subgroup analysis showed that EAP was superior to control group treatments (RR: 0.63; 95% CI: 0.50–0.79; *P*=0.005; Figure 7).

**Figure 8:**
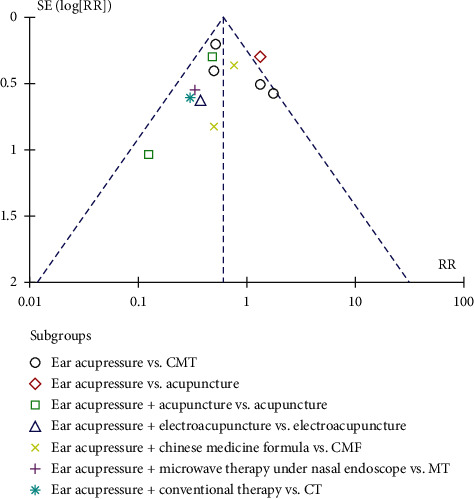
Funnel plot of total effective rate.

The funnel plot (Figure 8) suggested that publication bias may exist; however, other factors could also be present in Rao et al. (2005). This may due to poor design, in particular, the poorly allocated concealment method (the allocation concealment method was not mentioned in this study), leading to exaggerated treatment effects.

### 4.3. Secondary Outcomes

#### 4.3.1. Total Nasal Symptom Score

**Figure 9:**
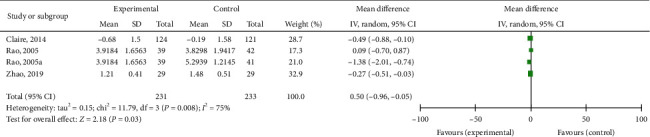
Forest plot for the total nasal symptom score.

**Figure 10:**
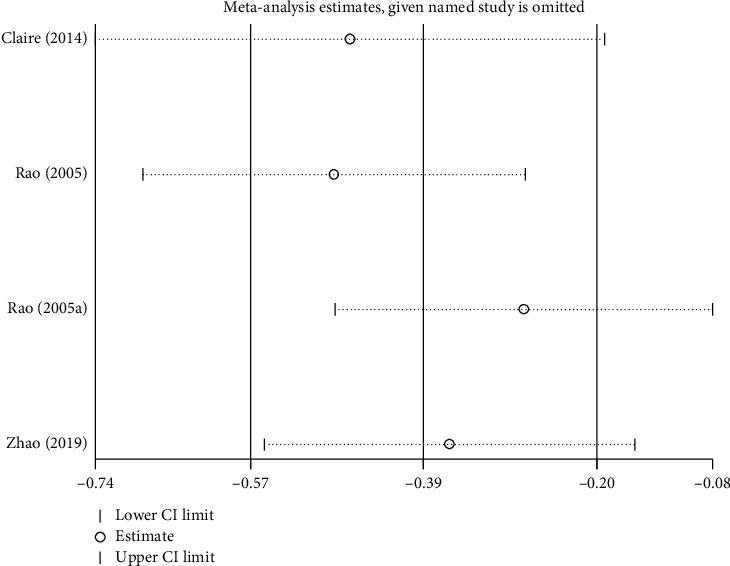
Sensitivity analysis for the total nasal symptom score.

Three studies reported the total nasal symptom score [32, 34, 45]. We pooled these data. We found that EAP was better than control group interventions (RR: −0.50; 95% CI: −0.96–0.05, *P*=0.03; Figure 9), including sham EAP, acupuncture, Western medicine cetirizine, and Shetizhiqiu decoction. There was significant heterogeneity among studies (*I*^2^ = 75%, *P*=0.008). Sensitivity analysis indicated that their result was robust (Figure 10).

#### 4.3.2. Runny Nose Score

**Figure 11:**
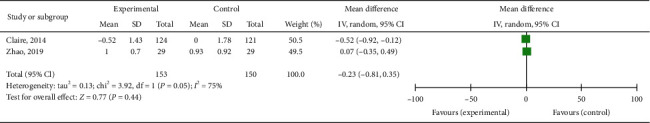
Forest plot for the runny nose score.

**Figure 12:**
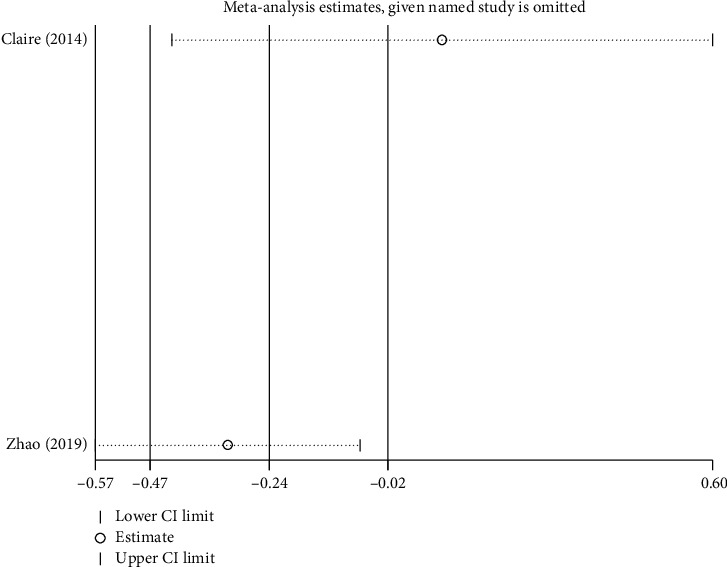
Sensitivity analysis for the runny nose score.

There were two trials noting the runny nose score [32, 45]. When EAP was compared with sham EAP or Shetizhiqiu decoction, the results were not statistically significant (RR: -0.23; 95% CI: -0.81–0.35; *P*=0.44; Figure 11). Sensitivity analysis indicated that this result was robust (Figure 12).

#### 4.3.3. Sneezing Score

**Figure 13:**
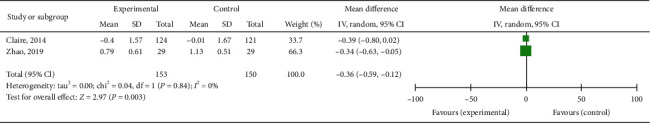
Forest plot for Sneezing score.

Two studies presented the sneezing score as outcome [39, 44]. Patients treated by EAP are superior to the patients in the control group (RR: −0.36; 95% CI: −0.59–0.12; *P*=0.003; Figure 13).

#### 4.3.4. Global QOL Score and Eye Symptom Score

Only Claire et al., 2014, used the eye symptom score and global QoL score. EAP was better than sham EAP in terms of the global QOl score improvement (RR: 0.42; 95% CI: 0.04–0.08; *P*=0.03) and eye symptom score (RR: −0.36; 95% CI: −0.67–0.05; *P*=0.02).

#### 4.3.5. EAP Related Adverse Events (AEs)

In these 12 studies, five did not mention adverse events information [35, 36, 38–40], and six trials [32, 34, 37, 41–43] recorded adverse events. Of these, three studies [37, 41, 45] reported adverse events in two groups. Li et al. reported that five participants in the control group had nasal mucosa drying and bloody nasal mucus [42]. In the study by Rao et al. [33], the incidence of adverse reactions was reported as follows: no adverse reactions occurred in the acupuncture group (2.17% ear pressure group) or the control group (13.04%). Statistical analysis showed that there was a significant difference between the control group and the ear pressure group (*P*=0.05). In the acupuncture group, a patient had dizziness and nausea during the process of acupuncture. After the needle was released quickly, it was completely relieved after resting for half an hour. There were six cases of mild or transient adverse reactions, including two cases of mild headache and drowsiness, four cases of dry mouth, and three cases of gastrointestinal discomfort. However, how these effects were resolved was not noted.

In the study by Claire et al. [32], details of safety were noted specifically. There were eight participants in the real group who reported 17 AEs and nine participants in the sham group who reported 20 AEs (*x*^2^ = 0.01; *P*=0.76). Some EAP-related AEs such as pellets irritating skin (one and two in real/sham groups) and ear acupoint inflammation (two and one events in real/sham groups) were reported during the 1^st^ week. These events were effectively managed by refining the pressing techniques by the participants, without any medical assistance required. On the other hand, they reported that some participants reported headache or dizziness (11 and 14 events in the real/sham group) and insomnia (two events in the sham group).

Another study presented safety information; they reported that, after microwave therapy under nasal endoscope [32], there were seven complications in the control group (11.7%), including three cases of haemorrhage, two cases of nasal stenosis, and two cases of infection. Complications occurred in five cases (8.3%) in the group, including two cases of bleeding and infection each, and one case of nasal cavity stenosis. There was no significant difference in the incidence of complications between the two groups. They also noted that the treatment group had no obvious adverse reactions after auricular point pressing.

## 5. Discussion

### 5.1. Overview of Findings

To the best of our knowledge, there was only one previous systematic review published in 2010 on this topic [32]. Five studies were included in the previous review. Three of those were excluded by us because diagnostic criteria were absent in the original texts (Tables 3 and 4). By detailed comparison, we discovered that Rao and Han (2006) [50] in their review was Rao et al. (2005) [34] in our review. These two studies illustrate the same experiment, with the same data and same author, however, in different publication years. In other words, this was a repeated publication. We chose Rao et al. (2005) instead of Rao and Han et al. (2006) because of the more detailed test records including laboratory instrument details, dropout details, and adverse reactions details. Hence, we have two studies identical to those of the previous review [34, 36]. In the present review, we tried to update the topic based on the findings of the previous review.

We included 12 studies. The control group intervention can be classified in six categories: sham ear acupressure, conventional medicine therapy, acupuncture, electroacupuncture, Chinese medicine formula, and microwave therapy under nasal endoscope. EAP was not inferior to control group interventions (conventional medicine therapy, acupuncture, electroacupuncture, Chinese medicine formula, and microwave therapy under nasal endoscope) in terms of improving effective rate of allergic patients^,^ symptom. However, the data extracted from 11 Chinese trials had small sample sizes and poor quality measures, according to the methodology measurement. The real EAP group was significantly greater than the sham group in terms of changes of global QoL score, scores for total nasal symptom, runny nose, and eye symptoms.

Validated questionnaire and scales such as the Quality Of Life Questionnaire are tools used to evaluate the quality of life of AR patients. The visual analogue scale is used to assess the severity of symptoms of AR. However, each has an application in included studies. Others such as quality of life score and nasal symptom scores evaluation methods can measure melioration of AR severity or disability; however, they are not widely used, despite the fact that these scales are recommended by the 2015 Clinical Guidelines.

### 5.2. Potential Biases in the Review Process

No ongoing trials were found. The conclusion of this review was drawn from the 12 trials, comprising a limited number of participants. More studies and high-quality trials should be included in future reviews. In addition, 3 key points that may cause potential heterogeneity may be summarized as follows:

As a noninvasive alternative, small seeds (Wang Bu Liu Xing and Vaccariae Semen) come from a plant or metal pellets on auricular points. Both are commonly used materials in EAP treatment and are effective. However, the differences between them remain unknown. In our review, Vaccariae Semen seeds applied in eleven China mainland publication trials [34–43, 45] and stainless steel pellets (1.2 mm in diameter; PELSST S/Steel Tan; Acuneeds Co., Camberwell, Victoria, Australia) were used in a two centres (Royal Melbourne Institute of Technology University (Melbourne, Australia) and Clinical Trial Clinic and Guangdong Provincial Hospital of Chinese Medicine, Guangzhou, China) (Claire et al.) [32]. This situation might make a subtle difference in terms of efficacy. More in-depth studies on the two raw materials may be needed.

Commonly used auricular points were summarized in our review: Nei Bi (TG4), lung (CO14), kidney (CO10), spleen (CO13), adrenal gland (TG2p), external nose (TG1, 2i), wind stream (SF1, 2i), and Shenmen (TF4). Therefore, these can be regarded as commonly used EAR for AR ear points. However, in Lu et al. (2015) [39], three ear acupoints including internal nose (TG4), sympathetic (AH6a), and root of ear tragus (R2) were selected. This is very different from other studies, which may be the source of heterogeneity, because EAP is based on the meridians theory of TCM. In meridians theory, each acupoint serves a different purpose. The specificity of acupoints in morphological structure, biophysical characters, pathological reactions, acupuncture stimulation-induced responses in different brain regions, and therapeutic effects were supported by scholars [44].

Despite our use of validated effectiveness assessment criteria documents supporting trials in this review, the nonuniform standard of efficacy evaluation might influence outcomes and results (especially effectiveness rate). It might be challenging to employ the same diagnosis and effectiveness assessment criteria for each trial, as these criteria vary with each update.

## 6. Conclusion

Despite the positive results of some outcomes, it is premature to confirm the efficacy of EAP for treating AR. More high-quality studies are needed to validate its efficacy. There are insufficient data to state that EAP is safe and reliable due to the small number of trials reporting adverse events. Therefore, studies with larger sample sizes and rigorously designed studies are necessary to determine conclusively a definitive association between EAP and AR.

## Figures and Tables

**Table 1:** Search strategy of the Cochrane Library.

Number	Search terms
1	Mesh descriptor: (ear acupressure) explode all trees
2	((Ear^*∗*^) or (acupressure^*∗*^) or (acupuncture^*∗*^) or (auricular^*∗*^) or (acupoint^*∗*^) or (sticking ^*∗*^)): ti, ab, kw
3	Or 1-2
4	Mesh descriptor: (allergic rhinitis) explode all trees
5	((Allergic rhinitis^*∗*^) or (allergic^*∗*^) or (rhinitis, allergic^*∗*^) or (rhinallergosis^*∗*^) or (AR^*∗*^) or (hay fever^*∗*^)): ti, ab, kw
6	Or 4–5
7	Mesh descriptor:(randomized controlled trials) explode all trees
8	(Random^*∗*^) or (randomly^*∗*^) or (allocation^*∗*^) or (random allocation^*∗*^) or (placebo^*∗*^) or (double blind^*∗*^) or (clinical trials^*∗*^) or (randomized control trial^*∗*^) or (RCT^*∗*^) or (controlled clinical trials^*∗*^)): ti, ab, kw
9	Or: 7-8
10	3 and 6 and 9

**Table 2:** Characteristics of 11 included studies.

Study ID	Sample size (E/C)	Male:female (E/C)	Age (E/C), year	Duration	Criteria of diagnose	Interventions		Period	Outcome measure
						Experimental group	Control group		
Yuan et al., 2013 [38]	120 (60/60)	Not given	3–14	≥1 year	Mentioned	Ear acupressure	Levocabastine	30 days	Effective rate, IgE, PAF

Li et al., 2018 [42]	72 (36/36)	19 : 17/16 : 20	18–65	Not given	Mentioned	Ear acupressure	Beclomethasone propionate nasal spray	3 weeks	Effective rate

Han et al., 2006 [35]	80 (40/40)	24 : 16/21 : 19	10–50	1–15 years	Mentioned	Ear acupressure	Astemizole	14 days/3 months	Effective rate

Rao, 2005 [34]	81 (39/42)	24 : 25/26 : 21	13–65	1–40 years	Mentioned	Ear acupressure	Acupuncture	1 month/follow-up	Effective rate, symptom score, IgE, TFN-*γ*

Rao and Han, 2005a [45]	80 (39/41)	24 : 25/25 : 21	13–65	1–40 years	Mentioned	Ear acupressure	Cetirizine tablets	1 month/follow-up	Effective rate, symptom score, IgE, TFN-*γ*

Claire et al., 2014 [32]	245 (124/121)	Not given	18–70	≥2 years	Mentioned	Real ear acupressure	Sham ear acupressure	8 weeks	Primary outcome measures: nasal and nonnasal symptom scores. Secondary outcomes:QoL score, change of relief medication usage, adverse events (AEs), and credibility of blinding

Ou et al., 2012 [37]	60 (30/30)	15 : 15/13 : 17	18–51/18–52	4–60 weeks	Mentioned	Ear acupressure + conventional therapy	Conventional therapy (cetirizine)	4 weeks	Effective rate, VAS

Ye et al., 2008 [36]	80 (40/40)	Not given	10–61	0.5–5 years	Mentioned	Ear acupressure + acupuncture	Acupuncture	4 weeks	Effective rate, symptom score

Yuan et al., 2016 [40]	120 (60/60)	27 : 33/28 : 32	21–67/19–69	2–11 years	Mentioned	Ear acupressure + acupuncture	Acupuncture	30 days	Effective rate

Lu et al., 2015 [39]	60 (30/30)	19 : 11/21 : 9	10–70	Not given	Mentioned	Ear acupressure + electroacupuncture	Electroacupuncture	1 week	Effective rate, RQLQ

Zhao, 2019 [43]	58 (29/29)	18 : 11/13 : 16	18–65	4.48 ± 1.9/4.24 ± 2.08	Mentioned	Chinese medicine formula + ear acupressure	Chinese medicine formula (Shetizhiqiu decoction)	4 weeks	Effective rate, symptom score

Liao, 2016 [41]	58 (30/28)	11 : 19/12 : 16	18–60	2.7 ± 2.26/1.86 ± 1.04	Mentioned	Chinese medicine formula + ear acupressure	Chinese medicine formula (Xinqin granule)	20 days	Effective rate

Li, 2017 [46]	120 (60/60)	62 : 58	15–74	1–18 years	Mentioned	Ear acupressure + microwave therapy under nasal endoscope	Microwave therapy under nasal endoscope	30 days	Effective rate

**Table 3:** Characteristics of the 25 excluded studies.

Study ID	Sample size (E/C)	Male:female (E/C)	Age (E/C), year	Duration	Criteria of diagnose	Interventions		Period	Outcome measure	Reasons of exclusion
						Experimental group	Control group			
Feng, 2020	70 (35/35)	20 : 15/22 : 13	10–49	4–16 years	Not mentioned	EAP + traditional Chinese medicine sticking	Cetirizine tablets	20 days	Effective rate	Diagnostic criteria reason and intervention reason

Zhou, 2015 [43]	60 (30/30)	14 : 16/13 : 17	18–60	Not mentioned	Mentioned	EAP + Chinese herbal medicine formula	Ebastine hydrochloride tablets	21 days	Effective rate	Intervention reason

Zeng, 2020 [30]	100 (50/50)	27 : 23/28 : 22	37–57	1–12 years	Not mentioned	EAP + Chinese medicine formula (Shenling Baizhu powder)	Cetirizine tablets	30 days	Effective rate; SF-36	Diagnostic criteria reason and intervention reason

Cai, 2019	36 (18/18)	10 : 8/11 : 7	19–55	Not mentioned	Mentioned	EAP + acupuncture + Chinese medicine formula (Wenyang Tongqiao formula)	Chinese medicine formula (Wenyang Tongqiao formula)	21 days	Effective rate	Intervention reason

Chen, 2015	70 (35/35)	20 : 15/18 : 17	15–70	1–22 years	Not mentioned	EAP + acupoint injection	Loratadine tablets + 1% ephedrine nasal drops	30 days	Effective rate:Total nasal symtom score	Diagnostic criteria reason and intervention reason

Chen, 2016	96 (48/48)	26 : 22/27 : 21	10–87	Not mentioned	Not mentioned	EAP + traditional Chinese medicine sticking + sublingual immunotherapy	Sublingual immunotherapy	30 days	Effective rate: total nasal symptom score	Diagnostic criteria reason and intervention reason

Cheng, 2011	60 (30/30)	19 : 11/20 : 10	17–59	1–20 years	Mentioned	EAP + massage	Beclomethasone propionate nasal aerosol	14 days	Effective rate: total nasal symptom score	Intervention reason

Lin, 2011 [8]	60 (30/30)	32 : 28	6–39	2–13 years	Mentioned	EAP + crude herb moxybustion	Acupuncture	Undefined	Effective rate	Intervention reason

Wang, 2012	80 (40/40)	22 : 18/25 : 15	11–71	1–25 years	Mentioned	EAP + massage	Cetirizine tablets	60 days	Effective rate: total nasal symptom score	Intervention reason

Wang, 2015	120 (60/60)	24 : 36/22 : 38	16–60	1–7 years	Mentioned	EAP + catgut in acupoint	Acupuncture	60 days	Effective rate	Intervention reason

Zhu, 2018	71 (48/29)	27 : 21/15 : 14	16–58	1.5–21 years	Mentioned	EAP + crude herb moxibustion	Azotyn tablets	2 weeks in the control group and 1 year on the treatment group	Effective rate; RQLQ	Intervention reason

Sun, 2019	60 (not mentioned)	30 : 25	8–62	1 month–15 years	Mentioned	EAP + Chinese medicine formula (Guizhi decoction)	EAP	20 days	Effective rate	Intervention reason

Xia, 2018	100 (50/50)	22 : 28/20 : 30	18–46	5 weeks–13 years	Not mentioned	Ear acupuncture + blood-letting therapy	Ear acupuncture	18 days	Effective rate	Diagnostic criteria reason and intervention reason

Li, 2013	100 (50/50)	22 : 28/20 : 30	19–49	1–10 years	Mentioned	EAP + sweet chrysanthemum capsule	Loratadine tablets	4 weeks	Effective rate: total nasal symptom score	Intervention reason

Xu, 2019	60 (30/30)	17 : 13/15 : 15	26–63	Not mentioned	Mentioned	EAP + Chinese medicine formula (Guizhi decoction)	Cetirizine tablets	Not mentioned	Effective rate: total nasal symptom score	Intervention reason

Huang, 2017	60 (30/30)	13 : 17/13 : 17	5–70	3weeks-7 years	Mentioned	EAP + Chinese medicine formula (Wenbi Tongqiao decoction)	Not mentioned	Not mentioned	Effective rate	Intervention reason

Xue, 2016	60 (30/30)	19 : 11/16 : 14	18–70	1–10 years	Mentioned	EAP + massage	Clarityne + Raynaud's nose spray	EAP: 1 days; massage: 6 months; Clarityne + Raynaud's nose spray: not mentioned	Effective rate: total nasal symptom score	Intervention reason

Fu, 2015	80 (40/40)	18 : 22/19 : 21	9–49/11–56	1 month-20 years	Not mentioned	EAP + Chinese medicine formula (Yupingfeng granule)	Azelastine nasal spray	2 weeks	VAS	Diagnostic criteria reason and intervention reason

Li, 2010	80 (47/33)	28 : 19/20 : 13	4–55/8–57	2–17 years	Mentioned	EAP + Chinese medicine formula (Yupingfeng granule)	Cetirizine tablets	20 days	Effective rate	Intervention reason

Lv, 2012	110 (60/50)	26 : 34/21 : 29	13–56/11–60	3 months–12 years	Mentioned	EAP + Chinese medicine formula (Yupingfeng granule)	EAP	3 months	Effective rate	Intervention reason

Wang, 2004	400 (300/100)	241/159	5–59	3 months–5 years	Not mentioned	EAP	Chinese medicine formula (Biyankang tablets)	1month	Effective rate	Diagnostic criteria reason

Kong, 2006	108 (54/54)	Not mentioned	14–62	Not mentioned	Not mentioned	EAP	Chinese medicine formula (Biyankang tablets)	Not mentioned	Effective rate	Diagnostic criteria reason

Huo, 2003	66 (30/36)	Not mentioned	22–65/20–62	Not mentioned	Not mentioned	EAP	Body acupuncture or antihistamine	Not mentioned	Effective rate	Diagnostic criteria reason

Rao, 2006 [45]	141 (42/39/41)	26 : 21/24 : 25/25 : 21	13–65	1–40 years	Mentioned	Ear acupressure	Acupuncture/cetirizine tablets	1 month/follow-up	Effective rate, symptom score, IgE, TFN-*γ*	Duplication

Li, 2017	120 (60/60)	62 : 58	15–74	1–18 years	Mentioned	Ear acupressure + microwave therapy under nasal endoscope	Microwave therapy under nasal endoscope	30 days	Effective rate	

**Table 4:** A list of excluded studies by reading full text.

Reason	Reference
Duplication (*n* = 1)	Rao Y.Q. and Han N.Y. (2006): therapeutic effect of acupuncture on allergic rhinitis and its effects on immunologic function (in Chinese). Zhongguo Zhen Jiu 26, 557–560

Diagnostic criteria (*n* = 4)	Wang W.H. (2004): 300 cases of allergic rhinitis treated by ear acupressure (in Chinese). Shanghai zhen jiu za zhi 23, 35
	Kong X.B., Ren H.Y., and Lu M.L. (2006): 108 cases of allergic rhinitis tested by ear acupressure (in Chinese). World Health Digest3, 34
	Huo Z.J. (2003): comparison of therapeutic effects of auricular acupuncture and body acupuncture on allergic rhinitis (in Chinese). Zhong Guo Zhen Jiu 23, 253–254

Intervention reason (*n* = 13)	Changqing L.: clinical observation of 60 cases of allergic F-inflammation treated by acupoint pressing with traditional Chinese medicine according to syndrome differentiation. Chinese community doctors 2012, 17 : 228–229. DOI: l0.3969/j.issn. l007 -6l4x.20l2.l7.2l7.
	Bofeng L.: observation on the curative effect of auricular point sticking and pressing plus yufeng powder on 47 cases of allergic rhinitis. Inner Mongolia traditional Chinese medicine 2010, 3 : 28–29. DOI: 10. 16040/j. cnki. cn15 -1101. 2010. 10. 045
	Haiyan X.: evaluation of the therapeutic efficiency of the comprehensive care combining auricular acupoint magnetic therapy and nasal acupoint massage on the prevention and treatment for the allergic rhinitis. Int J Nurs, June 2016, 35(11): 1569–1573.
	Huangjiali: auricular point pressing bean therapy was used to treat 60 cases of allergic rhinitis. Int J Nurs, June 2017, 3 : 259. Ying X.: efficacy of the Guizhi decoction plus auricular point on clinical symptoms in patients with allergic rhinitis. Clinical Journal of Chinese Medicine 2019, 32 (11):28–30.
	Xun L., LiXin T., Yali Z., and Jianhua L.: clinical observation of Xiangju capsule combined with auricular point pressing bean in the treatment of allergic rhinitis. Chinese Journal of Otolaryngology 2013, 12(1):23–25.
	LiSun: observation on the effect of guizhi decoction combined with allergic decoction and auricular point pressing therapy on allergic rhinitis. Contemporary Medical Symposium 2019, 17(2):187–188.
	Zhuhua: Crude herb moxibustion therapy combined with auricular point sticking therapy for allergic rhinitis. IMHGN, September 2018, 24(18):2751–2753. DOI: 10.3760/cma.j.issn.1007–1245.2018.18.007
	Quanquan W., Huimin H., Fang Z., Hailin C., and Maosen Z: observation of 40 cases of perennial allergic rhinitis treated by ear pressure combined with massage. Hebei J TCM 2012, 34(3):403–404.
	Yanxia L., Lihong D., and Liying F: observation on the curative effect of tian moxibustion plus auricular acupoint on allergic rhinitis. Journal of Medical Forum 2011, 32(2):183–184.
	Ling Z. and Wo Y.: clinical observation of the treatment of rhinitis 1 combined with auricular point platen press on allergic rhinitis. Heilongjiang Medical Journal 2015, 39(3):297–298.
	Hualei W. and Rong Y.: clinical observation of acupoint embedding line combined with ear pressure in the treatment of 60 cases of allergic rhinitis. Yunnan Journal of Traditional Chinese Medicine 2015, 36(12):57–58.
	Guolin C., Qiuhong Y., and Deyao C.: clinical observation on the treatment of allergic rhinitis by self-imitating five acupuncture points and four points combined with the warming yang tongqiao supplement-benefit method. Heilongjiang Medicine Journal 2019, 32(1):15–17.
	Zhipeng C., Li F., Jinming W., Lijun L., and Xiuxi T.: clinical observation of 30 cases of allergic rhinitis treated by massage and auricular point sticking. Journal of Community Medicine 2011, 9(1):49–50.
Diagnostic criteria reason and intervention reason (*n* = 6)	Shuyan F.: study on the value of auricular point pressing pill plus Chinese medicine sticking point in the treatment of allergic rhinitis. Broad vision of health 2020, 13 : 191.
	Jing C.: clinical observation on the treatment of allergic rhinitis by sublingual immunity combined with auricular point pricking and Chinese medicine application. Shenzhen Journal of Integrated Traditional Chinese and Western Medicine, 2016, 26(6):25–26.
	Zheng X.: to explore the clinical effect of ear acupuncture combined with blood-letting puncture in the treatment of allergic rhinitis. Feet and health care 2018, 12(194):177–178. DOI: 10.19589/j.cnki.issn 1004–6569.2018.12.17.7
	Fujixiong.: efficacy evaluation of auricular point sticking pressure combined with yufeng granules in treating allergic rhinitis. Journal of new medicine 2015, 47(2):189–190.
	Koufen C. and Xiuhua X.: observation on the effect of acupoint injection combined with auricular press in the treatment of perennial allergic rhinitis. Contemporary Medicine Forum 2015, 13(22):19–20.
	Huiyan Z.: clinical effect of Shenling Baizhu powder combined with ear acupressure pills on allergic rhinitis. China Modern Medicine 2020, 27(16):186–189.

Quasi-RCT (*n* = 1)	Lixia: effect of auricular point sticking combined with microwave under nasal endoscope in the treatment of allergic rhinitis. Journal of Chinese Rural Medicine 2017,24(20): 62–63.

## Data Availability

The data used to support the findings of this study are available from the corresponding author upon request.
